# Consumption of fermented dairy products is associated with lower anxiety levels in Azorean university students

**DOI:** 10.3389/fnut.2022.930949

**Published:** 2022-08-18

**Authors:** Rodrigo J. M. Sousa, José A. B. Baptista, Célia C. G. Silva

**Affiliations:** IITAA-Institute of Agricultural and Environmental Research and Technology, University of the Azores, Angra do Heroísmo, Portugal

**Keywords:** fermented food, anxiety, dietary intake, health, dairy, yogurt, STAI test

## Abstract

A growing number of studies have found that the gut microbiota is involved in a variety of psychological processes and neuropsychiatric disorders, which include mood and anxiety disorders. Consumption of dairy products may contain bioactive compounds and probiotic bacteria with various therapeutic benefits. The aim of the study was to investigate possible associations between the frequency of consumption of different types of dairy products and the state of anxiety in university students. The subjects were 311 Azorean university students, 231 women and 80 men, with an average age of 20.5 years. Subjects completed a quantitative questionnaire on the frequency of dairy product consumption and a short version of the Spielberger State-Trait Anxiety Inventory (STAI) test. Among dairy products, semi-skimmed milk was the most commonly consumed, followed by cheese (ripened), drinking yogurt, skim milk, and set yogurt, while fresh cheese, whole milk, and dairy ice cream were the least common. Discriminant analysis showed that consumption of fermented products (yogurt and cheese) was significantly higher (*P* < 0.05) in the group with low anxiety level (score <40 in STAI test) than in the group with higher anxiety level (score ≥ 40). In this analysis, 62.4% of the initially grouped cases were correctly classified according to the frequency of fermented products consumption. No correlations were found between anxiety and unfermented dairy products. The results indicate that the consumption of fermented dairy products has a positive effect on reducing anxiety in young Azorean university students.

## Introduction

Several studies in animals have shown that the commensal microbiota is crucial for the development of the hypothalamic-pituitary stress response (1–3). It has been shown that the absence of gut microbiota in germ-free animals is associated with an increase in risk behaviors and anxiety, which could normalize after gut colonization with bacteria from normal animals (4). In addition, observational studies in humans reported an altered gut microbiome in individuals with depression and depressive symptoms (5).

Components naturally present in the diet may play an important role in the viability, composition, and functionality of the gut microbiota (6). Recent evidence suggests that many aspects of the diet, such as its composition, consumption patterns, and cultural habits, have the potential to influence the interaction between the gut microbiome and the brain through multiple neuroendocrine pathways (7). In addition, there is growing evidence that the gut microbiome plays an active role in depression symptoms, anxiety, cognitive function, sleep, and brain function (8). Although there is still a need to fully understand these mechanisms, some evidence suggests that the gut microbiome may influence brain function through the excretion of metabolites, regulation of the host immune system, and ultimately the ability to secrete neuropeptides and active neurotransmitters (9). Due to the potential effect of probiotics on improving mental health, the term “psychobiotics” has been proposed (10). Psychobiotics refer to a group of probiotics that are able to produce and release neuroactive substances such as dopamine, norepinephrine, serotonin, and γ-aminobutyric acid (GABA), which act through the brain-gut axis and may exert antidepressant effects (11, 12). Current scientific evidence also suggests that lactic acid bacteria (LAB), particularly *Lactobacillus* (Firmicutes) and *Bifidobacterium* (Actinobacteria), help the host correct imbalances in the gut microbiota and consequently maintain and regulate mental health (9, 13). These bacteria are traditionally associated with fermented foods and are the most studied probiotic organisms (14). In this regard, consumption of fermented foods and probiotics have received considerable attention as potential treatments for depression and anxiety (15, 16).

Because fermented dairy products are an important source of probiotic microorganisms, we hypothesized in the current study that the state of anxiety may be influenced by the consumption of dairy products. A cross-sectional study was conducted to assess the association between dairy product consumption and the prevalence of anxiety in young university students.

## Materials and methods

### Participants

Participants were undergraduate students at the University of the Azores, Portugal. Participation was voluntary and informed consent was obtained before the start of the study. Of the 317 students who voluntarily participated in the survey, a total of 311 healthy students were included who did not meet the exclusion criteria of being over 40 years of age. Thus, the sample consisted of 311 young students of both sexes, 231 females and 80 males, with a mean age of 20.5 years (SD = 3.35). This study was approved by the Ethics Committee of the University of the Azores.

### Dairy food frequency questionnaire

The dairy intake survey was based on the Food Frequency Questionnaire (FFQ), adapted for the Portuguese population and validated by Lopes et al. (17). The questionnaire included 10 most common dairy foods and a frequency section with nine response options ranging from never to six or more times per day. Dairy product categories included milk (whole, semi-skimmed, and skimmed), yogurt (set and drinking yogurts), cheese (ripened cheese), fresh cheese (Latin-style cheese and whey-based cheese - requeijão, made without starter cultures), dairy desserts, and dairy ice cream.

### Spielberger state-trait anxiety inventory test

The short version of the Spielberger State-Trait Anxiety Inventory (STAI) was used to assess anxiety in university students. This test consists of six items, three of which are formed by questions about the presence of anxiety and three of which are formed by questions about the absence of anxiety (18). The short version (6-item version) of the STAI test was chosen instead of the 20-item STAI test because it is faster to use and the two tests give equivalent results (19). The scale for this test ranges from 20 to 80 points, with a higher score indicating greater anxiety. A cut-off of 39–40 has been proposed to detect clinically significant symptoms of anxiety for the STAI scale (20).

### Statistical analysis

The frequency of dairy products consumption was calculated using the validated questionnaires. Validation of the questionnaires was done by calculating Cronbach's alpha to determine the degree of reliability of the data obtained (21). A *t*-test for independent samples was performed to compare the consumption of dairy products between men and women. Discriminant analysis using Wilks' method was used to determine which dairy products provided significant discrimination between individuals exhibiting different levels of anxiety. Participants in the STAI test were divided into two groups according to the cutoff point at score 40 (20): group 1 - STAI score <40, corresponding to a normal/low anxiety state and group 2 - STAI score ≥40, corresponding to a high anxiety state. Dairy products were categorized as follows: milk consumption corresponded to all types of whole milk, semi-skimmed milk and skimmed milk; yogurts included set and drinking yogurts, Latin-style fresh cheese and whey cheese (locally called requeijão) were included in the “fresh cheese” group (non-fermented cheeses) and the last group included dairy ice cream and desserts. The pooled results from the consumption of yogurts (set and drinking) and ripened cheeses were classified as “fermented foods.” The assumptions of normality and homogeneity of the variance-covariance matrices of each group were tested using the Kolmogorov-Smirnov and M the Box tests, respectively. The association between anxiety according to the score group (low/normal and high anxiety) and consumption frequency of dairy foods, age, and sex was assessed using logistic regression with the forward stepwise method. *P* values <0.05 were considered statistically significant. Data were processed using the Statistical Package for the Social Sciences (SPSS IBM, USA, version 27).

## Results and discussion

### Assessment of ingestion of dairy products

The application of the dairy Food Frequency Questionnaire (FFQ) resulted in a total of 311 validated questionnaires. The average age for both sexes was 20.5 years, with a range between 17 and 37 years. Of the 311 respondents, 231 correspond to the female gender with a mean age of 20.5 years (SD = 3.6) and 80 correspond to the male gender with a mean age of 20.5 years (SD = 2.6). When analyzing the reliability of the questions constructed to evaluate the consumption of dairy products and dairy desserts, a Cronbach's α value of 0.7 was obtained, showing the correction of the internal consistency of the questionnaire according to Landis and Koch (21).

Table 1 shows the frequency of consumption of dairy products by the students. There was no significant effect of gender (*P* > 0.05) on the consumption of each dairy product. Almost all students (93.6%; female: 91.8%, male: 98.8%) consumed at least one serving of dairy products 2 to 4 times per week. Semi-skimmed milk was consumed most frequently (59.3%), followed by ripened cheeses (58.6%), drinking and set yogurts (39.8 and 34.1%, respectively), and skim milk (17.4%), while whey cheese, whole milk, and ice cream were consumed least (1.6, 2.5, and 8.3%, respectively). However, only half of the respondents (53.1%; women: 53.2%, men: 52.5%) consumed at least one dairy product per day. Among those who consumed at least one serving per day, semi-skimmed milk was the most consumed (32.6%), followed by ripened cheeses (15.2%), drinking and set yogurts (12.8 and 8.4%, respectively), and skim milk (9.4%).

**Table 1:** Frequency of consumption of dairy products among Azorean university students [*n* = 311, female = 231, male = 80].

**Frequency of dairy food consumption (%)**										
**Item**		**Never or < 1 ×month**	**1–3 ×month**	**1 ×week**	**2–4 ×week**	**5–6 ×week**	**1 ×day**	**2–3 ×day**	**4–5 ×day**	**≥6 ×day**
Full-fat milk	Female	96.1	1.7	0.0	0.4	0.0	1.3	0.4	0.0	0.0
	Male	95.0	1.3	0.0	1.3	2.5	0.0	0.0	0.0	0.0
	Total	95.8	1.6	0.0	0.6	0.6	1.0	0.3	0.0	0.0
Semi-skimmed milk	Female	29.9	8.2	5.2	18.2	6.9	17.7	10.4	1.3	2.2
	Male	22.5	7.5	3.8	23.8	7.5	18.8	13.8	0.0	2.5
	Total	28.0	8.0	4.8	19.6	7.1	18.0	11.3	1.0	2.3
Skimmed-milk	Female	71.4	7.8	1.7	5.6	3.9	6.1	2.2	0.9	0.4
	Male	81.3	3.8	2.5	1.3	2.5	3.8	3.8	1.3	0.0
	Total	74.0	6.8	1.9	4.5	3.5	5.5	2.6	1.0	0.3
Yogurt (set)	Female	27.7	19.9	18.2	20.3	6.5	5.6	1.7	0.0	0.0
	Male	31.3	21.3	13.8	18.8	3.8	10.0	1.3	0.0	0.0
	Total	28.6	20.3	17.0	19.9	5.8	6.8	1.6	0.0	0.0
Yogurt (drinking)	Female	28.1	19.0	14.7	16.9	9.5	10.0	1.3	0.0	0.4
	Male	28.7	18.8	7.5	23.8	5.0	11.3	3.8	0.0	1.3
	Total	28.3	19.0	12.9	18.6	8.4	10.3	1.9	0.0	0.6
Fresh cheese (Latin-Style)	Female	32.9	34.2	18.2	9.1	2.6	2.6	0.4	0.0	0.0
	Male	41.3	27.5	18.8	8.8	1.3	0.0	1.3	0.0	1.3
	Total	35.0	32.5	18.3	9.0	2.3	1.9	0.6	0.0	0.3
Wey cheese (Requeijão)	Female	85.3	10.4	2.6	1.7	0.0	0.0	0.0	0.0	0.0
	Male	88.8	8.8	1.3	0.0	1.3	0.0	0.0	0.0	0.0
	Total	86.2	10.0	2.3	1.3	0.3	0.0	0.0	0.0	0.0
Matured cheeses	Female	12.1	17.7	14.3	33.8	8.7	10.0	3.0	0.4	0.0
	Male	10.0	15.0	8.8	36.3	10.0	10.0	6.3	2.5	1.3
	Total	11.6	17.0	12.9	34.4	9.0	10.0	3.9	1.0	0.3
Dairy desserts	Female	24.7	46.3	15.6	10.4	1.7	1.3	0.0	0.0	0.0
	Male	22.5	51.2	16.3	3.8	2.5	1.3	1.3	0.0	1.3
	Total	24.1	47.6	15.8	8.7	1.9	1.3	0.3	0.0	0.3
Dairy ice-cream	Female	21.2	52.4	16.9	7.4	2.2	0.0	0.0	0.0	0.0
	Male	18.8	62.5	13.8	2.5	0.0	1.3	0.0	0.0	1.3
	Total	20.6	55.0	16.1	6.1	1.6	0.3	0.0	0.0	0.3

### Relationship of dairy products ingestion and anxiety

**Figure 1:**
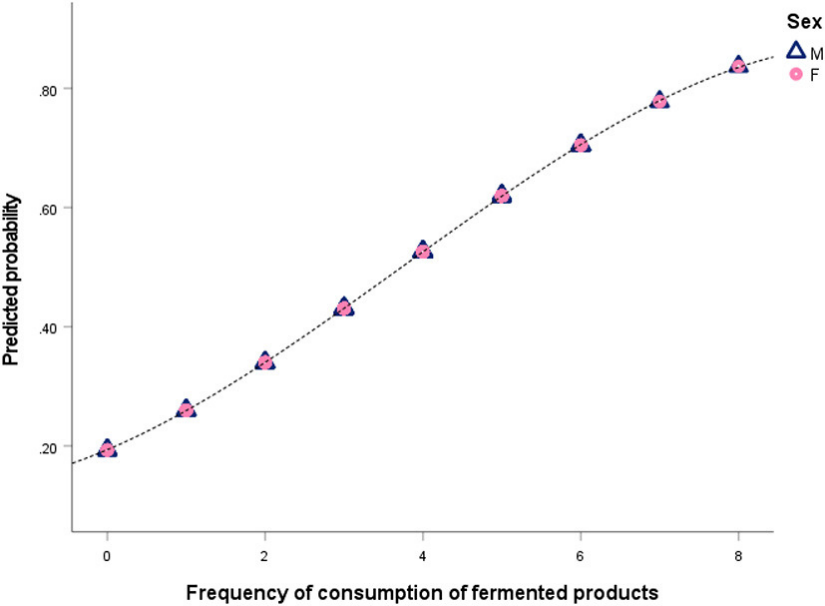
Predicted likelihood of having a normal anxiety state (STAI score <40) and consuming fermented foods (yogurt and cheese). Frequency of consumption of fermented products ranged from 0 (never or <1 × month) to 8 (≥ 6 × day), as indicated in Table 1. Males are represented by a blue triangle (M) and females (F) by a pink circle.

To assess possible associations between consumption of dairy products and state of anxiety, the STAI questionnaire was used. This questionnaire is most commonly used to assess anxiety in both psychiatric patients and the general population (22, 23). Marteau and Bekker (19) proposed a shorter STAI test with 6 items (STAI-6) that reliably replaces the longer questionnaire and can be used in behavioral research. The self-reported anxiety scores are shown in Table 2 and Figure 1 (Supplementary material). The STAI score ranges from 20 to 80, with a score of 34–36 considered normal. A higher score indicates severe anxiety, while a cutoff score of 39–40 has been suggested to identify clinically significant symptoms of state anxiety (20). The results showed that a high percentage of the Azorean college population scored above 40 on the STAI test, indicating high levels of anxiety in this population. The mean score was 40.5 ± 11.6, with 41.3 ± 12.2 in females and 38.4 ± 10.6 in males. Higher STAI scores were observed in females, consistent with other studies (23–25). The mean scores were also consistent with other studies conducted with college students (24, 25).

**Table 2:** Self-reported anxiety scores of the STAI-6 test of Azorean university students (*n* = 311).

	**Anxiety**	**Sex**		**Total**
		**Female**	**Male**	
Score	Average	41.3	38.5	40.6
	Standard error	12.3	10.7	11.9
Number of individuals	Normal (<40)	103	45	148
	High (≥40)	128	35	163
%	Normal (<40)	45.9	56.3	47.6
	High (<40)	55.4	43.7	52.4

*A score <40 was considered normal and a score ≥40 corresponds to an elevated anxiety level*.

Stepwise discriminant analysis extracted a discriminant function that retained the variables “yogurt” and “cheese” (ripened cheese) with significant discriminant power (*P* < 0.001). Table 3 shows the standardized coefficients of the yogurt and cheese variables in the discriminant function that explains the variability between the groups (group 1 - normal/low anxiety state and group 2 - high anxiety state). This function significantly discriminated the two groups (Λ = 0.930; X^2^ = 22.378; *P* < 0.001). Therefore, the consumption of fermented products (yogurt and ripened cheese) was higher in the group of subjects with normal anxiety state (score <40) than in the subjects with high anxiety state (STAI score ≥40; Table 4). The percentage of subjects correctly classified by discriminant analysis was 62.4%. Therefore, the consumption of fermented products, which include yogurt and ripened cheeses (fresh cheese without starter cultures excluded), may have a significant positive effect (*P* < 0.05) on the anxiety levels of the student respondents.

**Table 3:** Standardized coefficients of variables with discriminatory power (yogurt and aged cheese).

**Variables**	**Co-efficients in the discriminant function**
Yogurt	0.768
Cheese	0.571
*Eigenvalue*	0.077
Explained variance	100.0 %

**Table 4:** Ranking of discriminant analysis results for fermented foods consumed by subjects divided into two groups: STAI score <40 and STAI score ≥ 40.

	**Group**	**Predicted group membership^a^**		**Total**
		**<40**	**≥40**	
Number of individuals	Normal anxiety	88	67	148
	High anxiety	50	113	163
%	Normal anxiety	54,7	45,3	100,0
	High anxiety	30,7	69,3	100,0

a *62.4% of the original cases were correctly classified*.

Logistic regression with all predictors showed that age, sex, and consumption of dairy products (except fermented products) had no significant effect (*P* > 0.05) on the probability of having a normal anxiety level. In contrast, consumption of fermented products increased the probability of having a normal anxiety level (odds ratio, OR = 1.47, *P* < 0.05). Therefore, a new model was fitted with only the variable fermented products. The probability function for a normal/low anxiety state (STAI score <40) is shown in Figure 1.

There are few studies investigating the relationship between consumption of fermented foods and mental health. The present study confirms some evidence presented by other authors indicating the beneficial effects of fermented food consumption on the central nervous system (26–28). The mechanisms by which fermented foods affect mood can be explained in part by the production of neurotransmitters by certain microorganisms (29). Bacteria (e.g., *Lactobacillus* and *Bifidobacterium* species) associated with fermented foods may influence brain health through modulation of gut microbiota. Some gut microorganisms have been shown to alleviate anxiety and depression and improve cognitive performance (30). Moreover, mental disorders such as depression and anxiety are often associated with gut problems, suggesting a bidirectional relationship between mental health and gut microbiota (31).

Gamma-aminobutyric acid (GABA) production by lactic acid bacteria present in the gut and fermented foods has been proposed as one of the mechanisms involved (27, 28, 32). Fermented dairy products may contain probiotic microorganisms capable of surviving in the gastrointestinal environment and synthesizing GABA (26, 32). In the study by Luo et al. (33), ingestion of a GABA-producing strain of *Lactobacillus* was shown to reduce anxiety and improve cognitive function in animals with anxiety. Ingestion of milk fermented with a GABA-producing *Lactobacillus* strain has also been used to reduce anxiety and induce sleep in animals (28). In addition, several studies have shown that oral administration of GABA can affect the brain neurotransmitter system and improve symptoms of anxiety and depression (6, 32, 34–36). Other studies indicated that oral administration of selected probiotic bacteria may have beneficial effects in the treatment of gastrointestinal and psychological stress-related disorders (37–39). Recent human studies have shown that taking a dietary supplement containing probiotics, prebiotics, and phytobiotics (phytonutrients with gut-enhancing effects) improves beneficial gut bacteria and psychological well-being (40).

In conclusion, the present study demonstrates the association between the consumption of fermented dairy products such as yogurt and cheese (with the exception of fresh cheese) and lower anxiety levels in young university students. The results of this study should be considered in light of its limitations. Compared with other studies that investigated the eating behaviors of university students, the results of the present study are based on a relatively small sample size (311). Although the analysis was conducted with a small sample size, the homogeneity of the group in terms of age and ethnicity is a strength of the study. However, the use of university students may limit the generalizability of the results to the general population.

## Data availability statement

The raw data supporting the conclusions of this article will be made available by the authors, without undue reservation.

## Ethics statement

The studies involving human participants were reviewed and approved by Ethics Committee of the University of the Azores. The patients/participants provided their written informed consent to participate in this study.

## Author contributions

RS and CS acquired the data, conducted the data analyses, conceived, and designed the study. All authors contributed to the interpretation of data, drafted the manuscript, and approved the final version for publication.

## Funding

This work was supported by the Fundação para a Ciência e Tecnologia (FCT) — Project UID/CVT/00153/2019. Direção Regional da Ciência e Transição Digital (DRCTD) — Project M1.1.a/008/Funcionamento/2020.

## Conflict of interest

The authors declare that the research was conducted in the absence of any commercial or financial relationships that could be construed as a potential conflict of interest.

## Publisher's note

All claims expressed in this article are solely those of the authors and do not necessarily represent those of their affiliated organizations, or those of the publisher, the editors and the reviewers. Any product that may be evaluated in this article, or claim that may be made by its manufacturer, is not guaranteed or endorsed by the publisher.
