# Supplementary material for: Consumption of fermented dairy products is associated with lower anxiety levels in Azorean university students
Source: Front Nutr. 2022 Aug 18;9:930949. doi: 10.3389/fnut.2022.930949 (PMC9434012; doi:10.3389/fnut.2022.930949)

Supplementary material

Figure 1. Distribution of STAI test values by age (years) in university students from the Azores.

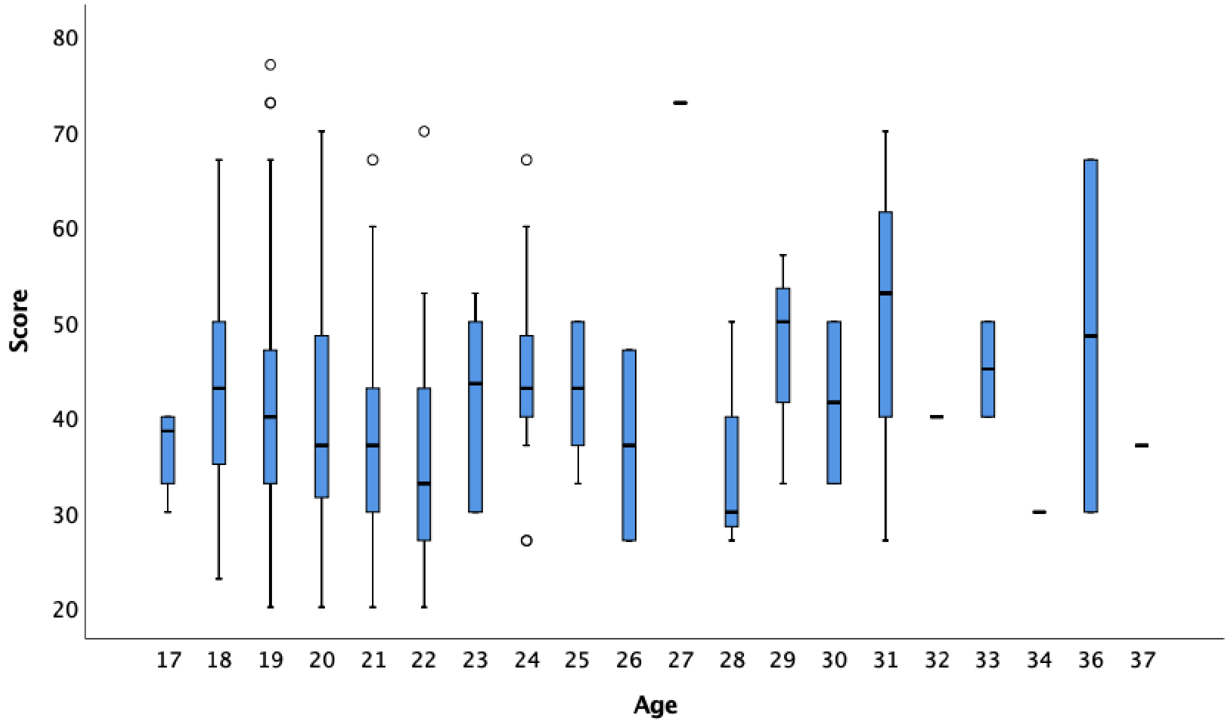

Supplement: Supplementary file 1 [file Image_1.PDF]
